# Supplementary material for: Mangrove consortium resistant to the emerging contaminant DEHP: Composition, diversity, and ecological function of bacteria
Source: PLoS One. 2025 Apr 24;20(4):e0320579. doi: 10.1371/journal.pone.0320579 (PMC12021221; doi:10.1371/journal.pone.0320579)
Supplement: Attachment — Submitted filename: Comment.docx [file pone.0320579.s003.docx]

Comments:

In this study, the authors isolated a bacterial consortium from neotropical mangrove using Di(2-ethylhexyl) phthalate (DEHP) as a stress source. They aimed to identify and characterize the DEHP-resistant microorganisms through gradient concentration exposure, DNA sequencing. The findings demonstrated that MP001 possesses a high potential for application in bioremediation purposes. However, significant concerns exists regarding the environmental significance and reasonability of the exposure design in its current form. Additionally, there are issues with the layout of figures and redundant/inaccurate information conveyed in some figures, which greatly compromises the readability of this paper.

The following specific points require further clarification and addressing:

1. In the Abstract, the authors mentioned the bacterial composition of exposure treatment and negative control. Are there any conclusion based on the changes in treatment and negative control groups?
2. The background and environmental context in which the study was conducted lack clarity. The selection of exposure doses ranged from 0.05 to 6 mg/L was based on specific considerations. However, there is a lack of available data or descriptions regarding the actual range of DEHP concentrations in the environment, which forms the core focus of this research.
3. In the present study, MP001 was isolated from the sediment of the Magé mangrove incubated at 37 ℃, and the exposure experiment was carried out at 35 ℃ ± 1. However, the average temperature in Rio de Janeiro state was reported as 17-36 ℃. Why did the authors choose such high temperatures? Does it really make sense to conduct bacteria isolation and exposure at these temperature?
4. In this study, the sediment sample was collected from the Magé mangrove in Rio de Janeiro state, which seems that the sediment sample was sampled at only one sampling site in the Magé mangrove. I’m worrying about the representativeness of this study because of the low sample size. If not, please provide the sampling sites of the study.
5. Could the media used for the separation and culture have a certain effect on bacteria determination? Why the media employed to bacteria isolation and exposure experiment were different?
6. The authors demonstrated that DEHP could provide as a carbon resource and degraded by the MP001 consortium in Line 313-314, Page 13. However, DEHP concentrations after exposure were didn’t determine. Thus, I consider this conclusion is unreasonable.
7. In Discussion section, the authors explain the dominant role of ARGs in xenobiotics resistance. However, it seems that the ARGs identification was absent in the present study.Therefore, I consider many elements of these paragraphs to be redundant and irrelevant to the content of this paper.
8. Ensure consistency in the significance in italics across the manuscript. Besides, several mistakes, such as “DEH Psediment” in Line 352, Page 14, disordered graphs (Figure 2 and 3)......
9. The authors should check and correct the mistakes in references according to the format requirement of the journal.
10. Several journal abbreviations need to be fixed and standard journal title abbreviations should be used throughout.
