# Supplementary material for: Mangrove consortium resistant to the emerging contaminant DEHP: Composition, diversity, and ecological function of bacteria
Source: PLoS One. 2025 Apr 24;20(4):e0320579. doi: 10.1371/journal.pone.0320579 (PMC12021221; doi:10.1371/journal.pone.0320579)
Supplement: Attachment — Submitted filename: Comments.docx [file pone.0320579.s005.docx]

Paper “Mangrove consortium resistant to the emerging contaminant DEHP: Composition, diversity, and ecological function of bacteria (PONE-D-24-45060)” is rewritten according to suggestions of distinguished reviewers and resubmitted. My suggestions and requests were properly addressed. Therefore, the revised paper could be accepted.
